# Comparative Analysis of Host Cell Entry Efficiency and Neutralization Sensitivity of Emerging SARS-CoV-2 Lineages KP.2, KP.2.3, KP.3, and LB.1

**DOI:** 10.3390/vaccines12111236

**Published:** 2024-10-30

**Authors:** Nianzhen Chen, Katharina Emma Decker, Sebastian R. Schulz, Amy Kempf, Inga Nehlmeier, Anna-Sophie Moldenhauer, Alexandra Dopfer-Jablonka, Georg M. N. Behrens, Metodi V. Stankov, Luis Manthey, Hans-Martin Jäck, Markus Hoffmann, Stefan Pöhlmann, Prerna Arora

**Affiliations:** 1 Infection Biology Unit, German Primate Center—Leibniz Institute for Primate Research, 37077 Göttingen, Germany; nchen@dpz.eu (N.C.); kdecker@dpz.eu (K.E.D.); akempf@dpz.eu (A.K.); inehlmeier@dpz.eu (I.N.); amoldenhauer@dpz.eu (A.-S.M.); mhoffmann@dpz.eu (M.H.); 2 Faculty of Biology and Psychology, Georg-August-University Göttingen, 37073 Göttingen, Germany; 3 Division of Molecular Immunology, Department of Internal Medicine 3, Friedrich-Alexander University of Erlangen-Nürnberg, 91054 Erlangen, Germany; sebastian.schulz@uk-erlangen.de (S.R.S.); hans-martin.jaeck@fau.de (H.-M.J.); 4 Department of Rheumatology and Immunology, Hannover Medical School, 30625 Hannover, Germany; jablonka.alexandra@mh-hannover.de (A.D.-J.); behrens.georg@mh-hannover.de (G.M.N.B.); stankov.metodi@mh-hannover.de (M.V.S.); manthey.luis@mh-hannover.de (L.M.); 5 German Centre for Infection Research (DZIF), Partner Site Hannover-Braunschweig, 30625 Hannover, Germany; 6 Center for Individualized Infection Medicine (CiiM), 30625 Hannover, Germany

**Keywords:** SARS-CoV-2 lineages, ACE2 receptor interactions, antibody evasion

## Abstract

New SARS-CoV-2 lineages continue to evolve and may exhibit new characteristics regarding host cell entry efficiency and potential for antibody evasion. Here, employing pseudotyped particles, we compared the host cell entry efficiency, ACE2 receptor usage, and sensitivity to antibody-mediated neutralization of four emerging SARS-CoV-2 lineages, KP.2, KP.2.3, KP.3, and LB.1. The XBB.1.5 and JN.1 lineages served as controls. Our findings reveal that KP.2, KP.2.3, KP.3, and LB.1 lineages enter host cells efficiently and in an ACE2-dependent manner, and that KP.3 is more adept at entering Calu-3 lung cells than JN.1. However, the variants differed in their capacity to employ ACE2 orthologues from animal species for entry, suggesting differences in ACE2 interactions. Moreover, we demonstrate that only two out of seven therapeutic monoclonal antibody (mAbs) in preclinical development retain robust neutralizing activity against the emerging JN.1 sublineages tested, while three mAbs displayed strongly reduced neutralizing activity and two mAbs lacked neutralizing activity against any of the lineages tested. Furthermore, our results show that KP.2, KP.2.3, KP.3, and LB.1 lineages evade neutralization by antibodies induced by infection or vaccination with greater efficiency than JN.1, particularly in individuals without hybrid immunity. This study indicates that KP.2, KP.2.3, KP.3, and LB.1 differ in ACE2 interactions and the efficiency of lung cell entry and suggest that evasion of neutralizing antibodies drove the emergence of these variants.

## 1. Introduction

The COVID-19 pandemic and the current COVID-19 endemic have been characterized by the rapid evolution of SARS-CoV-2, resulting in the continuous emergence of variants and sublineages with enhanced transmissibility, immune evasion, and potential resistance to therapeutic interventions [1,2,3,4,5]. These phenotypes have been mainly linked to mutations in the viral spike (S) protein, which facilitates viral entry into cells and is the key target of the neutralizing antibody response. For entry, the S protein binds to the ACE2 receptor and, upon activation by a cellular protease, either cathepsin L or TMPRSS2 fuses viral and cellular membranes [6]. Mutations in the spike protein can alter ACE2 binding affinity and host cell entry efficiency, potentially influencing viral transmission and pathogenicity [1,7]. Additionally, mutations may reduce the effectiveness of neutralizing antibodies, including therapeutic monoclonal antibodies, posing challenges for treatment and prevention [8].

The emergence of the highly mutated Omicron variant BA.1 in the winter season of 2021 constituted a quantum leap in SARS-CoV-2 evolution [9]. A similar event occurred in the autumn of 2023 when circulation of a highly mutated BA.2 sublineage, BA.2.86, was detected [10,11]. Upon acquisition of a critical amino acid exchange within the S protein, L455S, which increased antibody evasion, the BA.2.86 descendant JN.1 became globally dominant causing a wave of new infections and COVID-19 cases [12,13,14,15].

**Figure 1:**
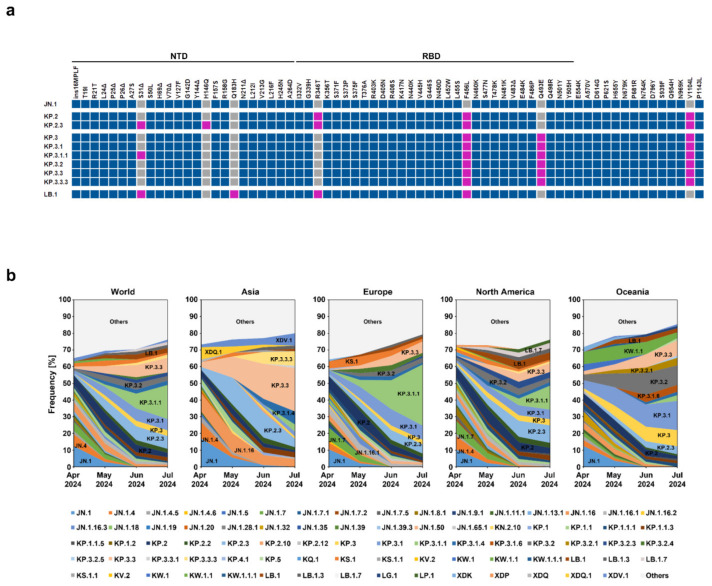
Spike protein mutations and global frequency of JN.1 sublineages (**a**) Mutations in the S proteins of SARS-CoV-2 lineages JN.1, KP.2, KP.2.3, KP.3, KP.3.1, KP.3.1.1, KP.3.2, KP.3.3, KP.3.3.3, and LB.1 compared to the S protein of the Wuhan-01 isolate. The mutations highlighted in pink indicate those that are unique for KP.2, KP.2.3, KP.3, KP.3.1, KP.3.1.1, KP.3.2, KP.3.3, KP.3.3.3, and LB.1 lineages compared to the JN.1 lineage. S protein mutations that are absent in certain SARS-CoV-2 lineages are highlighted in grey, while those mutations that are found in all lineages under study are highlighted in blue. Abbreviations: NTD, N-terminal domain; RBD, receptor-binding domain; (**b**) global frequencies of contemporary SARS-CoV-2 lineages. Lineages with frequencies below 1% were grouped together (labelled as “Others”). Data were retrieved from https://cov-spectrum.org/ (accessed on 7 August 2024) and represent mean frequencies that were calculated based on a seven-day sliding window.

Subsequently, JN.1 sublineages KP.2, KP.2.3, KP.3, and LB.1 have emerged that harbor lineage-specific mutations (Figure 1a) [16,17]. However, the impact of these mutations on host cell entry is unknown and the impact on antibody-mediated neutralization is incompletely understood [16,17].

Here, we report that all JN.1 sublineages enter target cell lines with appreciable efficiency and in an ACE2-dependent manner, with KP.3 being particularly adept at entering Calu-3 lung cells. The entry of all variants was ACE2-dependent, but evidence for differential ACE2 interactions was obtained. Finally, all variants evaded neutralizing antibodies with higher efficiency than JN.1.

## 2. Materials and Methods

### 2.1. Cell Cultures

The following cell lines were utilized in this study: BHK-21 (derived from Syrian hamster kidney cells; catalog number CCL-10, RRID, provided by Georg Herrler), Caco-2 (from human intestine; HTB-37, ATCC, RRID: CVCL_0025), Calu-3 cells (human, male, lung; HTB-55, RRID, provided by Stephan Ludwig), Vero cells (from female African green monkey kidney; catalog number CRL-1586, RRID, provided by Andrea Maisner), and 293T cells (from female human kidney; ACC-635, RRID), 293T human Cathepsin L knockout (293T_CTSL KO) (ab266521, Abcam, Cambridge, United Kingdom), and 293T human ACE2 knockout (293T_ACE2 KO) cells were created using the CRISPR-Cas9 system. For this, two single guide RNAs (sgRNA 1: AACATCTTCATGCCTATGTGAGG, sgRNA 2: TGCACAGAGAATATTCAAGGAGG) were cloned into the pLentiCRISPR v2 plasmid (Addgene #52961). For vector preparation, the plasmid was co-transfected with VSV-G and HIV-gag-pol into 293T cells. After 48 h, the supernatants were collected and used to transduce fresh 293T cells, followed by puromycin selection. Single cells were then isolated in 96-well plates, expanded into colonies, and ACE2-knockout confirmed via immunoblot analysis. All cell lines were maintained in Dulbecco’s Modified Eagle Medium (DMEM) sourced from PAN-Biotech (Aidenbach, Germany), enriched with 10% fetal bovine serum (FCS) from Biochrom (Berlin, Germany), and penicillin and streptomycin solution (P/S; PAN-Biotech) at final concentrations of 100 U/mL and 0.1 mg/mL, respectively. In addition, 293T_CTSL KO cells and 293T_ACE2 KO received 0.5 µg/mL and 1 µg/mL of puromycin respectively. For the cultivation of Calu-3 cells and Calu-3 cells that were engineered to stably express the beta-galactosidase omega fragment (referred to as Calu-3-Omega [18]), DMEM/F-12 medium (Thermo Fisher Scientific, Waltham, MA, USA) containing 10% FCS, P/S solution, non-essential amino acid solution (NEAA, 1:100 *v*/*v* dilution; PAN-Biotech), and 1 mM sodium pyruvate (PAN-Biotech) was used. The culture medium for Calu-3-Omega cells was further supplemented with puromycin at a concentration of 1 µg/mL (Invivogen; San Diego, CA, USA). For the cultivation of Caco-2 cells, minimum essential medium (GIBCO) containing 10% FCS, P/S solution, non-essential amino acid solution (NEAA, 1:100 *v*/*v* dilution; PAN-Biotech), and 1 mM sodium pyruvate (PAN-Biotech) was used. We incubated all cell lines at 37 °C in a humidified atmosphere with 5% CO_2_. For quality control, we screened all cell lines routinely for the absence of mycoplasma contamination. Furthermore, cell lines were authenticated by analysis of short tandem repeats (STR), sequencing of a part of the cytochrome c oxidase gene, phenotypic properties (examined via light microscopy), and/or cell line-specific growth characteristics. Transfection of cell lines was done by phosphate precipitation method (293T cells) or liposome-based transfection (BHK-21). For the latter, we used Lipofectamine 2000 (Thermo Fisher Scientific), per the manufacturer’s instructions.

### 2.2. Plasmids

We retrieved information on the different SARS-CoV-2 lineages and spike protein mutations from the GISAID (Global Initiative on Sharing All Influenza Data) and CoV-Spectrum (https://cov-spectrum.org/) databases (accessed on 7 August 2024). The SARS-CoV-2 S protein expression plasmids used for this study (summarized in Table 1) were generated by Gibson assembly. For this, we purchased five overlapping gene strings for each S protein (Thermo-Fisher Scientific). For assembly, the respective gene strings were mixed with linearized pCG1 plasmid (BamHI/XbaI-digested) and Gibson Assembly HiFi Master Mix (Thermo-Fisher Scientific), and incubated for 1 h at 56 °C. Thereafter, the mixtures were transfected into One Shot™ OmniMAX™ 2 T1R chemically competent Escherichia coli bacteria (Thermo-Fisher Scientific) and positive clones were subsequently identified by colony PCR. Finally, we confirmed the correctness of the inserted sequences by Sanger sequencing, making use of a commercial service (Microsynth SeqLab; Göttingen, Germany). In addition to the aforementioned S protein expression plasmids, we further used the following expression plasmids that have been described before [6,18,19]: pCAGGS-DsRed, pCAGGS-VSV-G, pCG1-sol-ACE2-Fc, pQCXIP-human ACE2-cMYC, pQCXIP-raccoon dog-ACE2-cMYC, pQCXIP-pangolin ACE2-cMYC, pQCXIP-civet ACE2-cMYC, pQCXIP-cat ACE2-cMYC, pQCXIP-mouse ACE2-cMYC, pQCXIP-*Rhinolophus affinis* ACE2-cMYC, pQCXIP-*Rhinolophus sinicus* ACE2-cMYC, pQCXIP-beta-galactosidase alpha fragment, and pQCXIP-beta-galactosidase omega fragment. The empty pCG1 expression plasmid was generously provided by Roberto Cattaneo.

### 2.3. Cell–Cell Fusion Assay

293T cells were transfected with either S proteins or an empty vector along with the beta-galactosidase alpha fragment (=effector cells). At 16–18 h post-transfection, the cells were washed and resuspended in fresh culture medium. The resuspended 293T cells were then added onto Calu-3-Omega cells, which stably express the beta-galactosidase omega fragment (=target cells). The co-cultures were incubated for 18 h to allow for S protein-driven cell-cell fusion. To assess the efficiency of cell-cell fusion, beta-galactosidase substrate (Gal-Screen, Thermo Fisher Scientific; Waltham, MA, USA) was added, followed by 90 min incubation. Cell lysates were then transferred to white-bottom plates, and luminescence was measured using a Hidex Sense plate luminometer (Hidex; Turku, Finland).

### 2.4. Production of VSV Pseudoparticles (VSV_pp_)

The 293T cells were transfected using calcium phosphate precipitation with pCG1 expression vectors encoding various S proteins, pCAGGS-VSV-G (positive control) or pCAGGS-DsRed (negative control). The transfected cells were then inoculated with VSV-G transcomplemented VSV*∆G (FLuc), a kind gift from Gert Zimmer [20]. Following one hour of incubation at 37 °C in 5% CO_2_ atmosphere, the supernatant was carefully removed and washed with phosphate-buffered saline (PBS). Subsequently, anti-VSV-G antibody (supernatant of I1-hybridoma cells; ATCC no. CRL-2700)-containing medium was supplemented to all cells except those expressing VSV-G. After incubating for an additional 16–18 h, the supernatant was collected and cell debris was removed by centrifugation (4000× *g*, 10 min). Lastly, the clarified supernatant was either used for experiments or stored at −80 °C for later use.

### 2.5. Transduction of Target Cells

Target cells were seeded in 96-well plates to assess the entry of pseudovirus particles. In order to investigate whether the S proteins of the here investigated variants are able to utilize human ACE2 or ACE2 orthologues from specific animals as receptors, BHK-21 cells were transfected to express the ACE2 orthologues (or none as control). The next day, the medium was changed following the transduction with pseudotype particles in equal volumes. Transduced cells were then incubated for 16–18 h at 37 °C with 5% CO_2_. In order to analyze the inhibition of cell entry of pseudotype particles by ammonium chloride, 293T or 293T_CTSL KO cells were treated with medium containing either 50 mM ammonium chloride (Sigma Aldrich; St. Louis, MO, USA) or medium without ammonium chloride as a control for 1 h at 37 °C before being transduced with pseudotype particles. For experiments aiming to investigate the dependency on TMPRSS2 on S protein-mediated cell entry, Caco-2 cells were treated with different concentrations of camostat mesylate (Sigma Aldrich; St. Louis, MO, USA) or DMSO as control. After 1 h at 37 °C, cells were transduced with pseudotype particles harboring indicated S protein variants. Efficiency of viral entry was assessed by measuring the activity of the pseudotype particle encoded firefly luciferase. Therefore, 50 µL/well PBS containing 0.5% Tergitol (Carl Roth; Karlsruhe, Germany) was added to the cells. After an incubation of 30 min at room temperature, in order to lyse the cells, the cell lysates were transferred to white 96-well plates. Equal volumes of luciferase substrate (Beetle-Juice, PJK; Kleinblittersdorf, Germany) were added to the wells and luminescence signal was measured using a Hidex Sense plate luminometer (Hidex; Turku, Finland).

### 2.6. Immunoblot

Particles containing the S protein of the analyzed variants (or no S protein as control) were concentrated by centrifugation at 25,000× *g* for 90 min at 4 °C through a 20% (*w*/*v* in PBS) sucrose cushion. Next, the concentrated particles were lysed by incubation with 2× sample buffer (0.06 M Tris-HCl, 20% glycerol, 4% SDS, 5% beta-mercaptoethanol, 0.4% bromophenol blue, 2 mM EDTA) for 15 min at 96 °C, before they were subjected to SDS-PAGE. Proteins were then blotted onto nitrocellulose membranes (Hartenstein; Würzburg, Germany) and following blocking of the membranes with PBS-T (PBS with 0.05% Tween 20, Carl-Roth; Karlsruhe, Germany) containing 5% bovine serum albumin (BSA, Carl-Roth; Karlsruhe, Germany) for 30 min at room temperature, the membranes were incubated with primary antibody (diluted in PBS-T with 5% BSA) overnight at 4 °C. For detection of SARS-CoV-2 S proteins, anti-SARS-CoV-2 (2019-nCoV) Spike S2 anti-body (rabbit, diluted 1:1000 in PBS-T with 5% BSA; SIN-40590-T62, Biozol; Eching, Germany) was used, while the loading control, vesicular stomatitis virus matrix protein (VSV-M) was detected using anti-VSV-M [23H12] antibody against the VSV matrix protein (VSV-M) as a loading control (mouse, diluted 1:1000 in PBS-T with 5% skim milk powder; EB0011, Kerafast; Shirley, MA, USA). Following primary antibody incubation, membranes washed three times for 10 min at room temperature with PBS-T and incubated for 1 h with anti-rabbit IgG (H+L)-HRPO (diluted 1:2000 in PBS-T with 5% skim milk powder; 111-035-003, Dianova; Eching, Germany) or anti-mouse IgG (H+L)-HRPO (diluted 1:2000 in PBS-T with 5% skim milk powder; 115-035-045, Dianova; Eching, Germany) for detection of SARS-CoV-2 S protein or VSV-M, respectively. After three additional washing steps with PBS-T, the respective proteins were visualized by applying a homemade chemiluminescence solution onto the membrane (0.1 M Tris-HCl [pH 8.6], 250 µg/mL luminol, 0.1 mg/mL para-hydroxy-coumaric acid, 1% H_2_O_2_) and signals were recorded using the ChemoCam imaging system including the ChemoStar Professional software (version 1.54d, Intas Science Imaging Instruments; Göttingen, Germany).

### 2.7. ACE2 Binding Efficiency

In order to assess the ACE2 binding efficiency of the SARS-CoV-2 S proteins under study, 293T cells were transfected with S protein expression plasmids or empty vector (control). Following an incubation period of 48 h, the cells were washed and resuspended in PBS containing 5 mM EDTA. Next, the cells were pelleted by centrifugation (300× *g* for 5 min at room temperature), resuspended in FACS buffer (PBS containing 2% BSA, 2 mM EDTA, and 0.2% NaN_3_) supplemented with soluble human ACE2-Fc at a dilution of 1:50 (*v*/*v*), and incubated for 45 min at 4 °C. Following this, the cells were washed and incubated with Alexa Fluor-488-conjugated anti-human antibody (diluted 1:200 in PBS-B; A-11013, Thermo Fisher Scientific; Waltham, MA, USA) for 45 min at 4 °C. Then, the samples were centrifuged at 300× *g* for 5 min at room temperature. Following aspiration of the supernatant, the cells were resuspended in FACS buffer and centrifuged again (300× *g* for 5 min at room temperature) in order to wash the cells. After discarding the supernatant, the cell pellets were resuspended in FACS buffer, before ACE2 binding was assessed with the help of the ID7000 Spectral Cell Analyzer (equipped with the ID7000 software version 1.1.8.18211; Sony Biotechnology; San Jose, CA, USA).

For the production of soluble human ACE2-Fc, 293T cells were transfected with plasmid pCG1-sol-ACE2-Fc and incubated for 48 h before the supernatant was collected, cleared from cellular debris by centrifugation, and concentrated 100-fold using VivaSpin^®^20 columns (30,000 MWCO, Sartorius, Göttingen, Germany).

### 2.8. Ethics Committee Approval and Enrollment of Study Participants

The collection and analysis of plasma samples were conducted as part of the COVID-19 Contact (CoCo) Study (DRKS00021152), approved by Hannover Medical School’s Internal Review Board (No. 8973_BO_K_2020). This prospective observational study monitors IgG and immune responses in healthcare professionals and individuals with potential SARS-CoV-2 contact at Hannover Medical School. Participants provided informed consent and received no compensation.

### 2.9. Plasma Samples

Samples were collected from two cohorts. The first cohort (cohort 1) entailed vaccinated individuals that received the XBB.1.5-adapted mRNA vaccine from Pfizer/Biontech (Raxtozinameran) as a last vaccination and who did not report any prior SARS-CoV-2 infection, which was further verified by absence of anti-SARS-CoV-2 NCP IgG (EUROIMMUN, Lübeck, Germany). The second cohort (cohort 2) included vaccinated individuals who did not receive the XBB.1.5-adapted mRNA vaccine from Pfizer/Biontech but instead experienced at least one SARS-CoV-2 infection (none of the participants experienced a severe disease), the last of which occurring in the winter season of 2023/24 in Germany, a time at which JN.1 was the dominating SARS-CoV-2 lineage. For quantification of anti-SARS-CoV-2-S antibody titers, we employed the QuantiVac-ELISA kit (EUROIMMUN, Lübeck, Germany). Supplementary Table S1 provides detailed information related to the plasma samples.

### 2.10. Neutralization Assay

For experiments addressing the neutralizing capacity of monoclonal antibodies (mAbs), S protein-bearing pseudovirus particles harboring were mixed with different mAb concentrations (0.2 ng/mL to 2 µg/mL), incubated for 30 min at 37 °C, and subsequently added onto Vero cells. Likewise, for experiments addressing the neutralizing activity of antibodies present in blood plasma, S protein-bearing pseudovirus particles were mixed with serial dilutions (1:25 to 1:6400) of heat-inactivated (56 °C, 30 min) blood plasma, incubated for 30 min at 37 °C, and subsequently added onto Vero cells. Pseudovirus particles incubated with medium without mAb or plasma served as a control. The Vero cells were further incubated for 15–16 h at 37 °C and 5% CO_2_, before the activity of virus-encoded luciferase (indicator for S protein-driven cell entry) was quantified in cell lysates as described above. Using signals obtained for cells that were inoculated with pseudovirus particles that were not exposed to mAbs/plasma as reference (0% inhibition), we next calculated the mAb concentration/plasma dilution required for half-maximal inhibition (effective concentration 50, EC50 for mAbs; neutralizing titer 50, NT50 for plasma). This was achieved by applying a non-linear regression model in GraphPad Prism (version 6.07). For discrimination between neutralization–positive and –negative samples, we defined for mAbs a threshold of EC50 ≤ 5 µg/mL (2.5 times the highest mAb concentration tested) and for plasma a threshold of NT50 ≥ 6.25 (25% of the lowest plasma dilution tested).

### 2.11. Statistical Analysis

For data analysis, we used Microsoft Excel (Microsoft Office Professional Plus, version 2016) and GraphPad Prism version 6.07. Specifically, we assessed statistical significance with a two-tailed Student’s *t*-test with Welch correction, a two-way analysis of variance with Dunnett’s post-test, and the Friedman test with Dunn’s multiple comparison test in GraphPad Prism. Statistical significance was assumed for *p*-values of 0.05 or lower (*, *p* ≤ 0.05; **, *p* ≤ 0.01; ***, *p* ≤ 0.001), while no statistical significance was assumed for p-values higher than 0.05. Details on the statistical tests used can be found in the figure legends.

## 3. Results

### 3.1. KP.2, KP.2.3, KP.3 and LB.1 S Proteins Are Efficiently Cleaved and Mediate Robust Entry into 293T, Vero, and Caco-2 Cells

The SARS-CoV-2 lineages KP.2, KP.2.3, KP.3, and LB.1 each harbor distinct mutations in the spike (S) protein as compared to the ancestral JN.1 lineage. KP.2 is characterized by mutations R346T, F456L, and V1104L. KP.2.3 shares these mutations and exhibits additional changes, including S31del and H146Q. KP.3 retains the mutations F456L and V1104L, similar to KP.2, but lacks S31del and H146Q. Finally, LB.1 is distinguished by the mutations S31del, Q183H, R346T, and F456L, setting it apart from the other lineages (Figure 1a). Analysis of the frequency of detection of these variants from April 2024 to July 2024 showed that JN.1 is losing its global prevalence, being replaced by variants such as KP.2, KP.2.3, KP.3, and LB.1. Thus, KP.2.3, KP.3, and, particularly, KP.3.1.1 (harbors an additional mutation in the S protein, delta S31, relative to KP.3), are increasing globally, while KP.2 and LB.1 cases are decreasing (Figure 1b).

**Figure 2:**
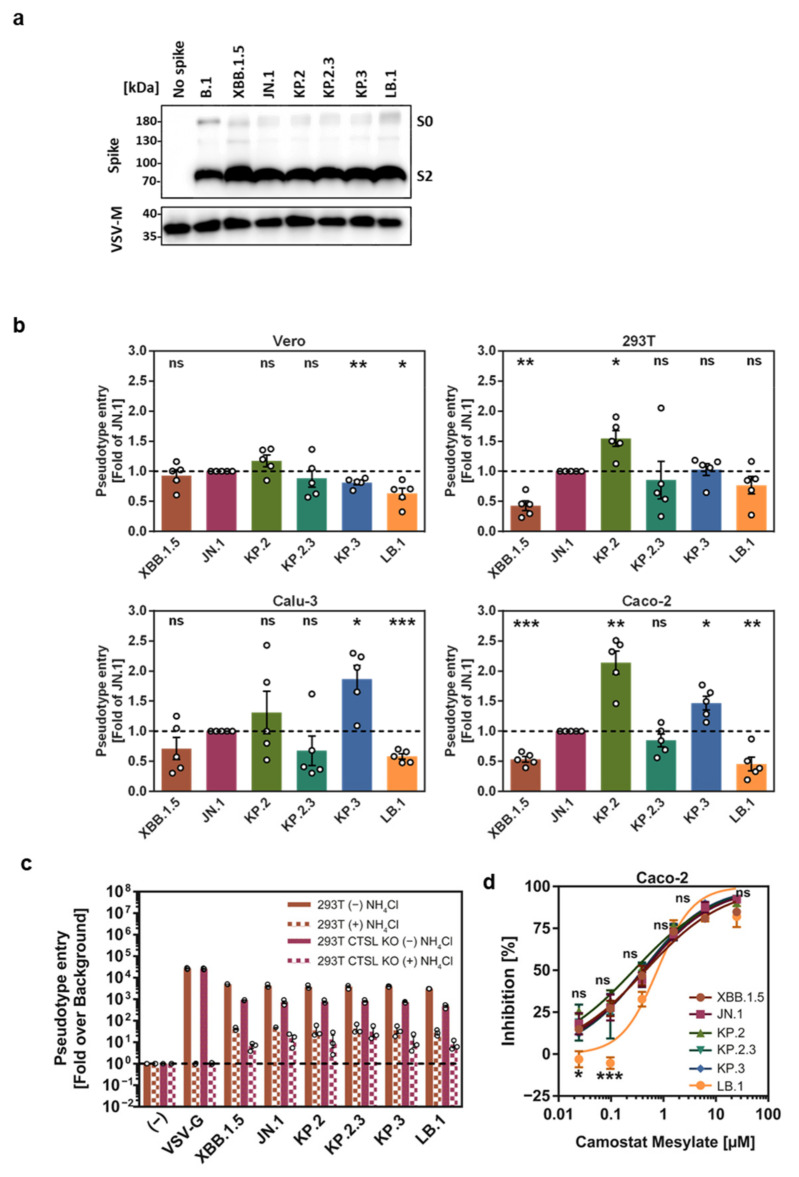
Cleavage and cell entry mediated by SARS-CoV-2 KP.2, KP.2.3, KP.3, and LB.1 S proteins. (**a**) Processing and pseudovirus particle incorporation of SARS-CoV-2 S proteins analyzed by SDS-PAGE and immunoblot (detection of vesicular stomatitis virus matrix protein (VSV-M) served as loading control). Presented are representative data from one out of three biological replicates. (**b**) Entry of pseudovirus particles bearing different SARS-CoV-2 S proteins into Vero (African green monkey, kidney), 293T (human, kidney), Calu-3 (human, lung), and Caco-2 (human, intestine) cells. Graphs show average (mean) entry data (normalized to signals obtained for particles bearing JN.1 S protein, set as 1) obtained from five biological replicates (four technical replicates per trial) with error bars indicating the standard error of the mean (SEM). See also Supplementary Figure S1a for additional information. (**c**) Entry of pseudovirus particles bearing different SARS-CoV-2 S proteins or VSV-G into 293T wildtype and 293T cathepsin L knockout cells in the presence and absence of ammonium chloride (50 mM). The graph shows average (mean) entry data (normalized to signals obtained for particles bearing no viral glycoprotein, set as 1) obtained from three biological replicates (four technical replicates per trial) with error bars indicating the SEM. (**d**) Entry of pseudovirus particles bearing different SARS-CoV-2 S proteins into Caco-2 cells in the presence of different concentrations of Camostat mesylate. The graph shows average (mean) entry data (normalized to signals obtained in the absence of Camostat mesylate, set as 0% inhibition) obtained from three biological replicates (four technical replicates per trial), with error bars indicating the SEM. Statistical significance was assessed by two-tailed Students’ *t*-test with Welch correction (panels b and c), or two-way analysis of variance (ANOVA) with Dunnett’s post-test (*p* > 0.05, not significant [ns]; *p* ≤ 0.05, *; *p* ≤ 0.01, **; *p* ≤ 0.001, ***).

In order to characterize host cell entry of the emerging JN.1 sublineages, we generated pseudovirus particles (_pp_) that harbored the KP.2, KP.2.3, KP.3, and LB.1 S proteins (KP.2_pp_, KP.2.3_pp_, KP.3_pp_, and LB.1_pp_) and examined particle incorporation, proteolytic processing, and S protein-mediated entry into cell lines. Particles bearing XBB.1.5 S protein (XBB.1.5_pp_) and JN.1 S protein (JN.1_pp_) served as controls. Immunoblot analysis confirmed that all S proteins were efficiently cleaved and incorporated into the pseudovirus particles (Figure 2a). For cell entry studies, we selected four cell lines that are commonly used to study host cell entry of SARS-CoV-2, which includes Vero (African green monkey, kidney) and 293T (Human, kidney), which allow only for SARS-CoV-2 S protein-driven by the cathepsin L-dependent endosomal entry route as they lack TMPRSS2 expression. Furthermore, we used Caco-2 (Human, colon) and Calu-3 (Human, lung) cells, which endogenously express the S protein-activating protease TMPRSS2 and thus allow for S protein-driven cell entry via the cell surface route. KP.2_pp_, KP.2.3_pp_, KP.3_pp_, and LB.1_pp_ entered Vero cells with similar efficiency to XBB1.5_pp_ and JN.1_pp_, although the entry of KP.3_pp_ and LB.1_pp_ was slightly reduced (Figure 2b). For 293T cells, the increased entry of JN.1_pp_, as compared to XBB.1.5_pp_, was observed, and KP.2 entered these with higher efficiency than JN.1_pp_. The entry phenotype observed with Caco-2 cells was similar to that detected for 293T cells, although the entry of KP.3 and LB.1_pp_ in the latter was significantly increased (KP.3_pp_) or reduced (LB.1_pp_) (Figure 2b and Supplementary Figure S1a). Finally, KP.3_pp_ entered Calu-3 cells with higher efficiency than JN.1_pp_, while the entry of LB.1_pp_ was reduced. In sum, KP.2.3_pp_ and LB.1_pp_ entered cell lines with the same or reduced efficiency, as compared to JN.1_pp_, while KP.2_pp_ and KP.3_pp_ showed a tendency for increased entry.

### 3.2. The S Proteins Studied Employ Cathepsin L and an Unknown pH Dependent Protease for Entry into 293T Cells While Caco-2 Cell Entry Is TMPRSS2-Dependent

We next investigated the dependence of cellular entry of KP.2_pp_, KP.2.3_pp_, KP.3_pp_, and LB.1_pp_ on the activity of the coronavirus S protein-activating cellular proteases cathepsin L (CTSL) and TMPRSS2 [6,21]. Dependence of entry on the activity of CTSL, a pH-dependent endo/lysosomal cysteine protease, was assessed using CTSL knockout 293T cells and NH_4_Cl treatment. CTSL knockout had no impact on VSV-G-driven entry but the pretreatment of cells with NH_4_Cl reduced entry to background levels, as expected (Figure 2c). Entry into 293T WT cells driven by all S proteins tested was reduced by the NH_4_Cl pretreatment of target cells, again in agreement with published data [6]. CTSL knockout also diminished entry but not to the same level as NH_4_Cl. Finally, the NH_4_Cl pretreatment of CTSL knockout cells diminished S protein-driven entry to levels slightly lower than those measured for pretreated WT cells, indicating that XBB.1.5 and JN.1 sublineages depend on CTSL and other pH-dependent proteases for entry into 293T cells. The ability of the JN.1 sublineages to use TMPRSS2 for entry was examined using Caco-2 cells and camostat mesylate, a serine protease inhibitor active against TMPRSS2 [22]. Camostat mesylate inhibited the Caco-2 cell entry of all pseudoparticles tested with similar efficiency, with the exception of LB.1_pp_ which showed increased Camostat sensitivity (Figure 2d). Thus, with the exception of LB.1_pp_, no substantial differences in CTSL and TMPRSS2 dependence were observed among the JN.1 sublineages.

### 3.3. Evidence for Differential ACE2 Interactions of KP.2, KP.2.3, KP.3, and LB.1 S Proteins

**Figure 3:**
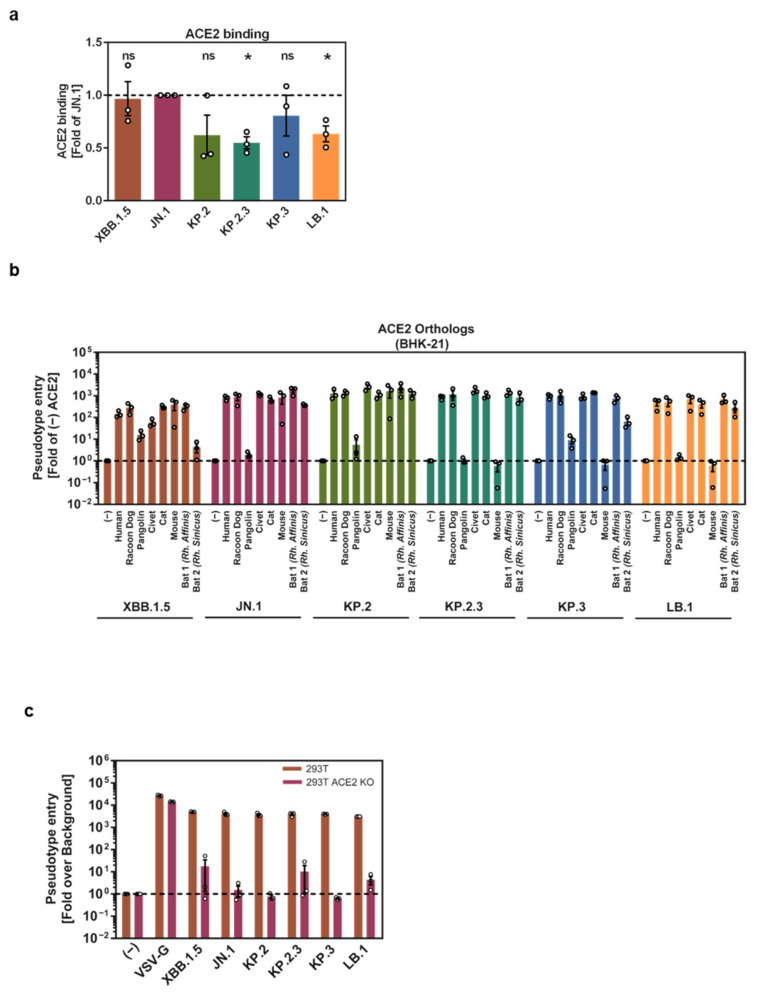
ACE2 usage of KP.2, KP.2.3, KP.3, and LB.1. (**a**) Binding of soluble human ACE2-Fc to 293T cells expressing SARS-CoV-2 S proteins. The graph shows average (mean) ACE2 binding data (normalized to signals obtained for JN.1 S protein, set as 1) obtained from three biological replicates (single samples per trial) with error bars indicating the SEM. Statistical significance was assessed by the two-tailed Students’ *t*-test with Welch correction (*p* > 0.05, not significant [ns]; *p* ≤ 0.05, *). See also Supplementary Figure S1b for additional information. (**b**) Entry of pseudovirus particles bearing different SARS-CoV-2 S proteins into BHK-21 cells transiently expressing mammalian ACE2 orthologues. The graph shows average (mean) entry data (normalized to signals obtained in the absence of ACE2 expression, set as 1) obtained from three biological replicates (four technical replicates per trial), with error bars indicating the SEM. (**c**) Entry of pseudovirus particles bearing different SARS-CoV-2 S proteins or VSV-G into 293T wildtype and 293T ACE2 knockout cells. The graph shows average (mean) entry data (normalized to signals obtained in the absence of ACE2 expression, set as 1) obtained from three biological replicates (four technical replicates per trial) with error bars indicating the SEM.

Subsequently, we assessed the ability of the KP.2, KP.2.3, KP.3, and LB.1 S proteins to interact with the SARS-CoV-2 receptor, ACE2. Flow cytometric analysis of the binding of S protein-expressing 293T cells to soluble human ACE2 (comprising the ACE2 ectodomain fused to the Fc portion of human immunoglobulin G) revealed that XBB.1.5, JN.1, and KP.3 S proteins bound to soluble human ACE2 with similar efficiency, while the binding of KP.2.3 and LB.1 S proteins was significantly reduced (Figure 3a). The ACE2 binding of KP.2 S protein was also reduced but this effect was not statistically significant (Figure 3a and Supplementary Figure S1c). More notably, the S proteins differed in their capacity to use species orthologues of ACE2 for cell entry. Employing BHK-21 cells transiently expressing ACE2 orthologues, we found that XBB.1.5 S, JN.1 S, and KP.2 S efficiently used mouse ACE2, whereas KP.2.3, KP.3, and LB.1 S did not (Figure 3b). Furthermore, KP.3 S protein, unlike the other five S proteins tested, utilized Chinese horseshoe bat (*Rhinolophus sinicus*) ACE2 only with low efficiency for entry. These results point towards differences in the ACE2 interactions of the S proteins tested. Finally, we employed 293T cells in which endogenous ACE2 expression was knocked out via CRISPR/Cas9 to determine if entry of the variants under study was ACE2-dependent. The absence of ACE2 expression had no appreciable impact on entry driven by VSV-G but reduced entry mediated by all S proteins tested close to or within background levels (Figure 3c). Signals marginally above background were measured for JN.1_pp_, KP.2.3_pp_, and LB.1_pp_, but these effects were not statistically significant (Figure 3c). In sum, KP.2 S, KP.2.3 S, and LB.1 S bound to ACE2 with slightly reduced efficiency as compared to JN.1 S, and evidence for differences in the mode of ACE2 engagement among the S proteins of the emerging JN.1 sublineages was obtained.

### 3.4. KP.2, KP.2.3, and LB.1 S Proteins Drive Cell–Cell Fusion with Reduced Efficiency as Compared to JN.1 S Protein

**Figure 4:**
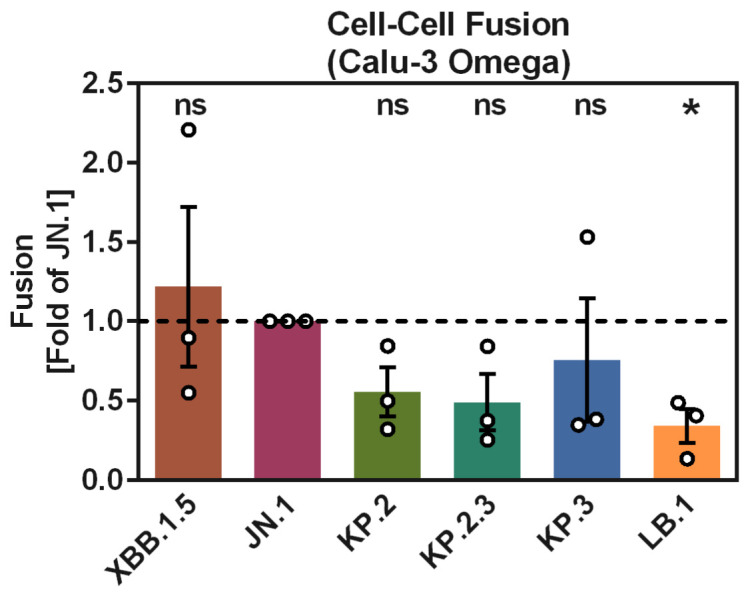
Cell–cell fusion induced by SARS-CoV-2 KP.2, KP.2.3, KP.3, and LB.1 S proteins. Fusion of 293T effector cells transiently expressing SARS-CoV-2 S proteins and the beta-galactosidase alpha fragment with Calu-3 Omega effector cells (Calu-3 cells stably expressing the beta-galactosidase omega fragment). The graph shows average (mean) entry data (normalized to signals obtained for JN.1 S protein, set as 1) obtained from three biological replicates (single samples per trial), with error bars indicating the SEM. Statistical significance was assessed by two-tailed Students’ *t*-test with Welch correction (*p* > 0.05, not significant [ns]; *p* ≤ 0.05, *). See also Supplementary Figure S1c for additional information.

The fusion of infected cells with uninfected cells is driven by the S protein and is believed to contribute to COVID-19 pathogenesis [23,24,25]. We evaluated the capacity of the KP.2 S, KP.2.3 S, KP.3, and LB.1 S proteins to induce cell–cell fusion, making use of a split beta-galactosidase reporter assay (Figure 4 and Supplementary Figure S1c). For this, we co-cultured 293T cells that were co-transfected with expression plasmids for the respective S protein and the beta-galactosidase alpha-fragment (=effector cells) with Calu-3 cells stably expressing the beta-galactosidase omega-fragment (Calu-3 Omega cells = target cells). Following the co-culture of effector and target cells, cell–cell fusion was quantified by measuring the activity of the reconstituted beta-galactosidase in cell lysates [18]. Cell–cell fusion driven by KP.2, KP.2.3, KP.3, and LB.1 S proteins was reduced compared to XBB.1.5 S and JN.1 S, and this effect was statistically significant for LB.1 S protein (Figure 4). Thus, the mutations in the S proteins of the emerging JN.1 sublineages reduce the capacity for cell–cell fusion.

### 3.5. KP.2_pp_, KP.2.3_pp_, KP.3_pp_, and LB.1_pp_ Are Highly Resistant Against Neutralization by Several Monoclonal Antibodies in Pre-Clinical Development

Recombinant monoclonal antibodies (mAbs) have been instrumental in COVID-19 therapy and prophylaxis. However, many current SARS-CoV-2 lineages have developed resistance to most or all clinically-approved mAbs [26]. Consequently, we focused on a set of pre-clinical antibodies BD57-0129, BD56-1302, BD56-1854 [27], Omi-42 [28], BD55-4637, BD55-5514, and BD55-5840 [29], which have demonstrated activity against the BA.2.86 variant [26]. BD55-4637, BD55-5514 (SA55), and BD55-5840 (SA58) have been isolated from vaccinated SARS convalescents [29]. In addition, BD56-1854 and BD56-1302 were isolated from patients infected by the BA.2 lineage, while BD57-0129 was isolated from a patient infected by the BA.5 lineage [27]. Finally, Omi-42 was isolated from a patient infected with the BA.1 lineage [28]. To assess the neutralizing potential of these mAbs, pseudovirus particles bearing the various S proteins were preincubated with serial dilutions of each mAb before being inoculated onto Vero cells. The subsequent reduction in infectivity, compared to control pseudoviruses not exposed to mAbs, was measured to determine the neutralizing capacity of each antibody.

**Figure 5:**
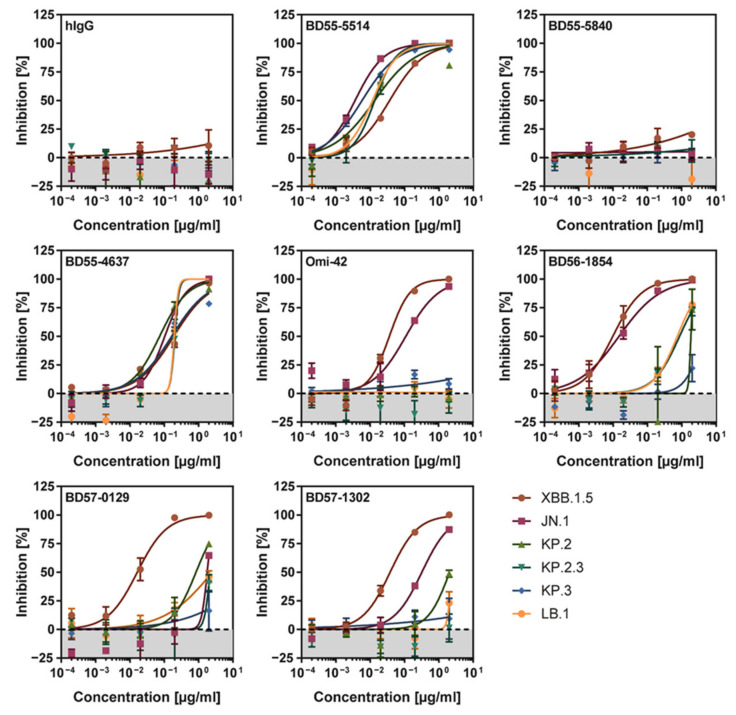
Neutralization of KP.2, KP.2.3, KP.3, and LB.1 by monoclonal antibodies. Pseudovirus particles bearing different SARS-CoV-2 S were preincubated with different concentrations of monoclonal antibody (mAb), were inoculated onto Vero cells, and cell entry was analyzed. The graph shows average (mean) entry data (normalized to signals obtained in the absence of mAb, set as 0% inhibition) obtained from three biological replicates (four technical replicates per trial), with error bars indicating the SEM.

Among the seven mAbs tested, five (BD55-5514, BD55-4637, Omi-42, BD56-1854, BD56-1302) efficiently neutralized XBB.1.5_pp_ and JN.1_pp_ (Figure 5). In contrast, KP.2_pp_ and LB.1_pp_ showed reduced sensitivity to neutralization by BD56-1854, while KP.2_pp_, KP.2.3_pp_, KP.3_pp_, and LB.1_pp_ were not, or were only marginally, neutralized by Omi-42, BD57-0129, or BD57-1302. Finally, BD55-5840 failed to neutralize any of the S protein-bearing pseudoviruses tested (Figure 5 and Supplementary Figure S2a), while BD55-5514 retained high neutralizing activity against all S protein analyzed. Collectively, KP.2_pp_, KP.2.3_pp_, KP.3_pp_, and LB.1_pp_ efficiently evaded most mAbs in pre-clinical development, with the exception of BD55-5514.

### 3.6. KP.2_pp_, KP.2.3_pp_, KP.3_pp_, and LB.1_pp_ Evade Neutralizing Antibodies Induced by Vaccination or Infection with Higher Efficiency than JN.1_pp_

**Figure 6:**
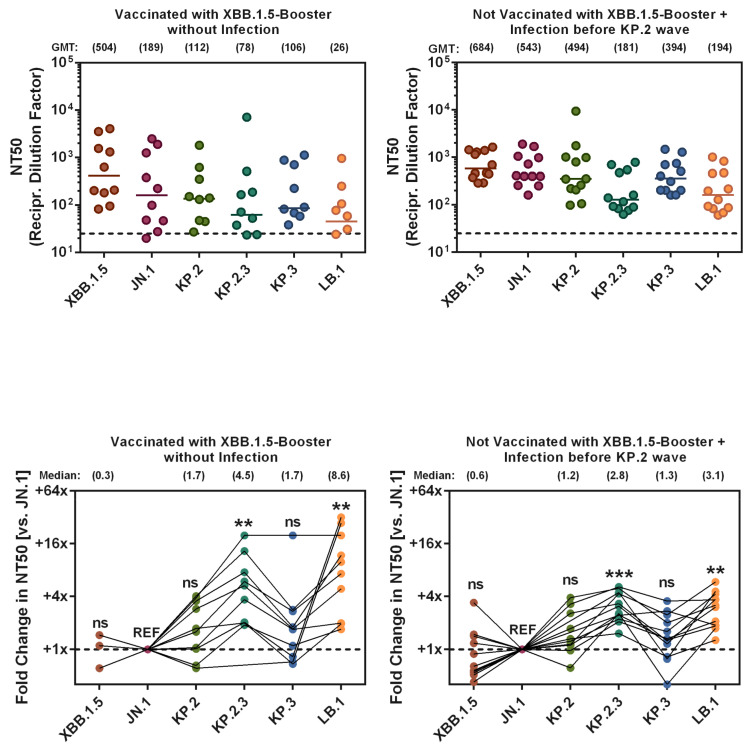
Neutralization of KP.2, KP.2.3, KP.3, and LB.1 by antibodies in the plasma of two cohorts with different immunization background: cohort 1, vaccinated including XBB.1.5-adapted booster vaccine/no history of SARS-CoV-2 infection (*n* = 10); cohort 2, vaccinated (no XBB.1.5-adapted booster vaccine/history of one (*n* = 6) or two SARS-CoV-2 infections (*n* = 6). Pseudovirus particles bearing different SARS-CoV-2 S were preincubated with different dilutions of human plasma were inoculated onto Vero cells and cell entry was analyzed. Next, plasma dilutions leading to half-maximal inhibition (neutralizing titer 50, NT50) were determined by normalizing cell entry of plasma-exposed pseudovirus particles to entry of particles in the absence of plasma (set as 0% inhibition). The graphs show geometric mean neutralization titers (GMT; top panels), with dashed lines indicating the lowest plasma dilution tested and normalized neutralization data for donor-matched plasma samples (relative to neutralization of pseudovirus particles bearing JN.1 S protein, set as 1; bottom panels). Data were obtained from a single experiment (four technical replicates). Information above the graphs indicate GMT values (top panels) or the fold change in neutralization compared to pseudovirus particles bearing JN.1 S protein (bottom panels). Statistical significance was assessed by the Friedman test with Dunn’s multiple comparison test (*p* > 0.05, not significant [ns]; *p* ≤ 0.005, **; *p* ≤ 0.001, ***). Of note, samples with NT50 <12.5 were defined as negative and assigned a value of 1. See also Supplementary Figure S1c for additional information. See also Supplementary Table 1 and Supplementary Figure S2 for additional information.

Finally, we evaluated the sensitivity of the emerging sublineages to neutralization by antibodies generated upon vaccination and/or prior infection, using blood plasma from two patient cohorts with different immunization histories. Cohort 1 consisted of individuals who had no prior history of SARS-CoV-2 infection and received the XBB.1.5-adapted mRNA vaccine as their most recent vaccination (*n* = 10). Cohort 2 included individuals who did not receive the XBB.1.5-adapted mRNA vaccine but had experienced one or two SARS-CoV-2 infections (Supplementary Table S1). Across all cohorts, neutralization of XBB.1.5_pp_ was most efficient, with geometric mean titers (GMT) ranging from 504 to 684, followed by the neutralization of JN.1_pp_ (GMT = 189–543) (Figure 6, Supplementary Figure S2b). Notably, the neutralization of the emerging lineages KP.2_pp_, KP.2.3_pp_, KP.3_pp_, and LB.1_pp_ was reduced when compared to XBB.1.5_pp_ and JN.1_pp_ (GMT = 26–494). Finally, the reduction in neutralization of LB.1_pp_ in cohort 1, and both LB.1_pp_ and KP.2.3_pp_ in cohort 2, was statistically significantly as compared to JN.1_pp_.

## 4. Discussion

Our findings demonstrate that the emerging SARS-CoV-2 lineages KP.2, KP.2.3, KP.3, and LB.1 enter cell lines efficiently and in an ACE2-dependent manner. Although the S proteins of these lineages robustly engage human ACE2, they display the differential utilization of ACE2 orthologues from animal species, suggesting substantial differences in ACE2 interactions and, potentially, differential potential to spill over to animals. Moreover, although we did not observe increased human ACE2 binding by the S proteins of SARS-CoV-2 lineages KP.2, KP.2.3, KP.3, and LB.1 compared to the JN.1 S protein, there may be still differences regarding their potential to interact with other attachment factors such as neuropilin-1, KREMEN-1, ASGR1, heparan sulfate, sialic acids, or TMEM106B [30,31,32,33,34,35,36,37,38,39]. This possibility requires further investigation. Finally, the KP.2, KP.2.3, KP.3, and LB.1 lineages were resistant against five out of seven monoclonal antibodies in pre-clinical development, which efficiently neutralized JN.1 and evaded antibodies induced by infection and/or vaccination with higher efficiency than JN.1.

The KP.2, KP.2.3, KP.3, and LB.1 S proteins were efficiently cleaved and incorporated into pseudovirus particles and facilitated entry into diverse cell lines. KP.2_pp_ and KP.3_pp_ exhibited similar or increased entry compared to JN.1_pp_, with the augmented entry of KP.3_pp_ into Calu-3 lung cells being particularly noteworthy, while LB.1_pp_ showed a tendency for reduced entry. However, since these results were obtained solely using pseudoviruses and cell lines, formal confirmation with authentic virus and primary respiratory cells and/or in vivo models is required.

We previously reported that BA.5_pp_ and BA.2.86_pp_ exhibited increased Calu-3 lung cell entry [26,40], which points towards the increased capacity to spread in lung tissue and cause severe disease. Indeed, BA.5 exhibited augmented spread in murine and ferret lung as compared to previously circulating Omicron subvariants [40], and a chimeric virus encoding BA.5 but not BA.2 S protein caused disease in hamsters [40,41]. Whether the capacity of KP.3 to spread in human lung tissue is increased as compared to KP.2, KP.2.3, and LB.1 remains to be investigated. Finally, we note that previous studies using HOS-ACE2-TMPRSS2 cells reported the reduced infectivity of KP.2_pp_ and KP.3_pp_, and similar infectivity of KP.2.3_pp_ and LB.1_pp_ as compared to JN.1_pp_ [17]. The discrepancy between these and our results are most likely due to differences in target cell choice, with HOS-ACE2-TMPRSS2 cells not being available for the present study.

Maintaining robust ACE2 interactions is important for transmissibility, as demonstrated for JN.1 S and XBB.1.5 S protein, which bound to human ACE2 with similar high efficiency, in keeping with published findings [12,18,42]. The binding of KP.2, KP.2.3, KP.3, and LB.1 S proteins to human ACE2 was slightly reduced as compared to XBB.1.5 and JN.1 S protein. Mutations such as F456L and L455S, present in KP.2, reduce hydrophobic contacts between the receptor-binding domain (RBD) [13] and residues T27, K31, D30, and H34 in ACE2, thereby diminishing ACE2 affinity, potentially contributing to reduced ACE2 binding. In contrast, the R346T mutation stabilizes the receptor-binding motif (RBM), and might allow for appreciable ACE2 binding despite the presence of mutations like F456L and L455S [13].

The KP.2 S protein but not the KP.2.3, KP.3, and LB.1 S proteins efficiently used murine ACE2 for cell entry. In the context of the S protein of the Beta variant mutations K417N and N501Y allowed for efficient cell entry via murine ACE2 [43] and these mutations are present in all JN.1 sublineages studied. As a consequence, additional mutations present in the S proteins of KP.2.3, KP.3, and LB.1 S proteins might be interfering with engagement of murine ACE2 and mutational analysis is needed to identify the responsible residues. Furthermore, differential usage of murine ACE2 and the reduced ability of KP.3 S protein to use ACE2 from *Rh. sinicus* points towards differences among JN.1 sublineages in the mode of ACE2 engagement that merit investigation within future studies. In this context, it needs to be stated that the latter data were obtained using transient overexpression of ACE2 orthologues and thus the observed phenotypes may differ when endogenous ACE2 expression is analyzed.

Several mAbs, including BD56-1854, Omi-42, BD57-0129, and BD56-1302 exhibited reduced or no neutralization activity against the emerging JN.1 subvariants tested. BD55-5850 exhibited no neutralization activity since its epitope is altered by the R346T mutation present in subvariants KP.2, KP.2.3 and LB.1 [29]. BD55-5514 was the only monoclonal antibody among the seven tested that efficiently neutralized all JN.1 sublineages tested. This antibody targets an epitope within the receptor-binding domain (RBD), which includes V503 and G504, residues that are critical for ACE2 binding [29]. Furthermore, the BD55-5514 epitope belongs to the F3 group, which comprises residues that are highly conserved among clade I sarbecoviruses [44], accounting for the broad neutralizing activity of BD55-5514.

The emerging SARS-CoV-2 lineages demonstrated greater neutralization evasion compared to JN.1, in keeping with two previous reports [16,17], particularly in individuals vaccinated with the XBB.1.5-adapted mRNA vaccine. Although augmented antibody evasion was only significant for KP.2.3 and LB.1, likely due to the small sample size, this finding suggests that augmented antibody evasion accounts at least in part of the success of these variants. Specific spike protein mutations likely contributing to antibody evasion in emerging SARS-CoV-2 lineages could include those located in the receptor-binding domain (RBD), such as R346T, F456L, and V1104L. Mutation R346T has been associated with immune evasion in other variants [27,45,46], and the presence of multiple mutations in KP.2, KP.2.3, and KP.3, and LB.1 likely contributes to their enhanced resistance to antibodies, especially in those vaccinated with XBB.1.5-adapted vaccines. Altogether, these findings highlight the ongoing challenge of immune evasion and the importance of broad-spectrum therapies. We wish to point out that our study has limitations. This includes a lack of data on the infection and replication of authentic virus in primary cells and in vivo models, the use of transient overexpression models to study ACE2 usage of SARS-CoV-2 S proteins, and the absence of data on potential differences in SARS-CoV-2 lineage-specific neutralization by antibodies induced upon vaccination with updated JN.1- or KP.2-based mRNA vaccines.

## 5. Conclusions

KP.2, KP.2.3, KP.3, and LB.1 evaded antibodies with increased efficiency as compared to JN.1, suggesting that immune evasion has been a major driver behind the emergence of these variants. Cell entry of these variants was ACE2-dependent, and the S proteins of all variants bound appreciably to human ACE2, although with slightly different efficiency. However, the marked differences in usage of species orthologues of ACE2 point towards differences in the mode of ACE2 engagement. Thus, KP.2 used murine and human ACE2 with comparable efficiency for cell entry, while the KP.2.3, KP.3, and LB.1 variants failed to appreciably use murine ACE2, suggesting that these variants might not be amenable to analysis in mouse models. Finally, it is noteworthy that KP.2 and KP.3, the two sublineages that bound to human ACE2 with roughly comparable efficiency as JN.1, were also the ones that entered cell lines with the same or increased efficiency as compared to JN.1. This suggests that, for these variants, efficient antibody evasion was compatible with robust ACE2 engagement.

## Figures and Tables

**Table 1:** Expression plasmids for SARS-CoV-2 S proteins.

**Expression** **Plasmid**	**SARS-CoV-2 Lineage**	**GISAID** **Identifier**	**Modifications**
pCG1-SARS-CoV-2 B.1 S∆18	B.1	EPI_ISL_425259	codon-optimized with a C-terminal truncation of last 18 amino acids
pCG1-SARS-CoV-2 XBB.1.5 S∆18	XBB.1.5	EPI_ISL_16239158	codon-optimized with a C-terminal truncation of last 18 amino acids
pCG1-SARS-CoV-2 JN.1 S∆18	JN.1	EPI_ISL_18530042	codon-optimized with a C-terminal truncation of last 18 amino acids
pCG1-SARS-CoV-2 KP.2 S∆18	KP.2	EPI_ISL_19197864	codon-optimized with a C-terminal truncation of last 18 amino acids
pCG1-SARS-CoV-2 KP.2.3 S∆18	KP.2.3	EPI_ISL_19197559	codon-optimized with a C-terminal truncation of last 18 amino acids
pCG1-SARS-CoV-2 KP.3 S∆18	KP.3	EPI_ISL_19203001	codon-optimized with a C-terminal truncation of last 18 amino acids
pCG1-SARS-CoV-2 LB.1 S∆18	LB.1	EPI_ISL_19067004	codon-optimized with a C-terminal truncation of last 18 amino acids

## Data Availability

Raw data are available upon request. This study did not generate code. All materials and reagents will be made available upon installment of a material transfer agreement.

## Supplementary Materials

The following supporting information can be downloaded at: https://www.mdpi.com/article/10.3390/vaccines12111236/s1, Figure S1: host cell entry, ACE2 binding, cell–cell fusion of the emerging JN.1 sublineages KP.2, KP.2.3, KP.3, and LB.1; Figure S2: neutralization sensitivity of the SARS-CoV-2 emerging KP.2, KP.2.3, KP.3, and LB.1 lineages; Table S1: plasma information.
