# Supplementary material for: Comparative Analysis of Host Cell Entry Efficiency and Neutralization Sensitivity of Emerging SARS-CoV-2 Lineages KP.2, KP.2.3, KP.3, and LB.1
Source: Vaccines (Basel). 2024 Oct 30;12(11):1236. doi: 10.3390/vaccines12111236 (PMC11598761; doi:10.3390/vaccines12111236)
Supplement: Supplementary file 1 [file vaccines-12-01236-s001.zip › SI_Figure legends.pdf]

Fig. S1. Host cell entry, ACE2 binding, cell-cell fusion of the emerging JN.1 sublineages KP.2, KP.2.3, KP.3 and LB.1.

(a) Host cell entry of SARS-CoV-2 lineages. Particles bearing the indicated S proteins, vesicular stomatitis glycoprotein (VSV-G, positive control), or no viral glycoprotein (negative control) were inoculated onto the indicated cell lines and entry was analyzed at 16–18 h post inoculation by measuring firefly luciferase activity in cell lysates. Presented are the mean data from five biological replicates, conducted with four technical replicates, and cell entry was normalized against the assay background (signals obtained from particles bearing no viral glycoprotein, set as 1). Error bars represent the SEM. (b) ACE2 binding. 293T cells expressing the indicated S proteins following transfection were incubated with soluble human ACE2-Fc and Alexa Fluor-488-conjugated anti-human antibody, before ACE2 binding was analyzed by flow cytometry. Presented are the mean fluorescence intensity (MFI) data from three biological replicates, conducted with a single technical replicate. Error bars indicate the standard deviation (SD). (c) Cell-cell fusion. Effector 293T cells transfected to express the indicated S protein along with the beta-galactosidase alpha fragment were coincubated for 18h with target Calu-3 cells stably expressing the beta-galactosidase omega fragment. Next, S protein-driven cell-cell fusion was analyzed by quantification of reconstituted beta-galactosidase activity in cell lysates. Presented are the mean data from four biological replicates, conducted with three technical replicates, and fusion was normalized against the assay background (signals obtained after coincubation of target cells with effector cells that did not express S protein, set as 1). Error bars indicate the SEM; Table S1: Plasma information.

Fig. S2. Neutralization sensitivity of the SARS-CoV-2 emerging KP.2, KP.2.3, KP.3 and LB.1 lineages

(a) Effective dose 50 (EC50) values calculated from the data presented in figure 5. (b) Individual neutralization data for plasma samples of the two cohorts. Presented are the mean data from one biological replicate, conducted with four technical replicates, and cell entry was normalized against particles incubated in the absence of plasma (set as 0% inhibition). Error bars indicate the SD.
