# Supplementary material for: Comparative Analysis of Host Cell Entry Efficiency and Neutralization Sensitivity of Emerging SARS-CoV-2 Lineages KP.2, KP.2.3, KP.3, and LB.1
Source: Vaccines (Basel). 2024 Oct 30;12(11):1236. doi: 10.3390/vaccines12111236 (PMC11598761; doi:10.3390/vaccines12111236)
Supplement: Supplementary file 1 [file vaccines-12-01236-s001.zip › Supplementary Table S1.pdf]

Table S1: Plasma information

General information						Vaccination status	Infection status		
Sample ID	Cohort	Gender	Age (years)	Days since last immunization[a]	IgG (BAU/ml)[b]	Vaccination history	Infected (Yes/No)	Date of infection(s)	Most prevalent lineage(s) at the time of infection
10170	1	Female	26	15	6627	V#1: Yes (n.i.); V#2: Yes (n.i.); V#3: Yes (n.i.); V#4: BNT (B.1/BA.5); V#5: BNT (XBB.1.5)	No	n.a.	n.a.
10179	1	Female	30	16	5110	V#1: BNT (B.1); V#2: BNT (B.1); V#3: BNT (B.1); V#4: BNT (B.1); V#5: BNT (XBB.1.5)	No	n.a.	n.a.
10182	1	Female	42	15	2125	V#1: BNT (B.1); V#2: BNT (B.1); V#3: BNT (B.1); V#4: BNT (B.1/BA.5); V#5: BNT (XBB.1.5)	No	n.a.	n.a.
10186	1	Male	74	15	1882	V#1: AZD; V#2: AZD; V#3: BNT (B.1); V#4: MOD (B.1); V#5: BNT (XBB.1.5)	No	n.a.	n.a.
10192	1	Female	48	16	3523	V#1: BNT (B.1); V#2: BNT (B.1); V#3: BNT (B.1); V#4: BNT (B.1/BA.5); V#5: BNT (XBB.1.5)	No	n.a.	n.a.
10197	1	Female	57	16	2531	V#1: BNT (B.1); V#2: BNT (B.1); V#3: BNT (B.1); V#4: BNT (B.1); V#5: BNT (B.1/BA.5); V#6: BNT (XBB.1.5)	No	n.a.	n.a.
10198	1	Female	64	16	6560	V#1: AZD; V#2: AZD; V#3: BNT (B.1); V#4: BNT (B.1); V#5: BNT (XBB.1.5)	No	n.a.	n.a.
10200	1	Male	57	16	2106	V#1: BNT (B.1); V#2: BNT (B.1); V#3: BNT (B.1); V#4: Yes (n.i.); V#5: Yes (n.i.); V#6: BNT (B.1); V#7: Yes (n.i.); V#8: BNT (XBB.1.5)	No	n.a.	n.a.
10215	1	Male	38	16	2202	V#1: BNT (B.1); V#2: BNT (B.1); V#3: BNT (B.1); V#4: BNT (B.1); V#5: BNT (XBB.1.5)	No	n.a.	n.a.

10223	1	Female	25	21	4461	V#1: AZD; V#2: AZD; V#3: BNT (B.1); V#4: BNT (B.1/BA.5); V#5: BNT (XBB.1.5)	No	n.a.	n.a.
10410	2	Male	31	44	2796	V#1: BNT (B.1); V#2: BNT (B.1); V#3: BNT (B.1)	Yes	27.12.2023	JN.1
10445	2	Female	56	79	4973	V#1: AZD; V#2: Yes (n.i.); V#3: Yes (n.i.); V#3: BNT (B.1/BA.5)	Yes	25.11.2023	JN.1, BA.2.86.1
10503	2	Female	64	47	1597	V#1: AZD; V#2: BNT (B.1); V#3: BNT (B.1); V#4: BNT (B.1/BA.5)	Yes	28.12.2023	JN.1
10543	2	Male	50	60	1522	V#1: BNT (B.1); V#2: Yes (n.i.); V#3: Yes (n.i.)	Yes	16.12.2023	JN.1
10620	2	Male	58	88	2074	V#1: AZD; V#2: BNT (B.1); V#3: BNT (B.1); V#4: BNT (B.1/BA.5)	Yes	19.11.2023	JN.1, BA.2.86.1
10642	2	Female	56	69	5541	V#1: BNT (B.1); V#2: Yes (n.i.); V#3: Yes (n.i.); V#4: BNT (B.1/BA.5)	Yes	09.12.2023	JN.1
10475	3	Female	58	54	5937	V#1: BNT (B.1); V#2: BNT (B.1); V#3: BNT (B.1)	Yes	12.03.2022<br>20.12.2023	BA.1, BA.2<br>JN.1
10504	3	Female	56	44-74	2226	V#1: BNT (B.1); V#2: BNT (B.1); V#3: BNT (B.1)	Yes	xx.08.2022<br>xx.12.2023	BA.5, BE.1.1<br>JN.1
10533	3	Female	57	78	2647	V#1: AZD; V#2: BNT (B.1); V#3: BNT (B.1)	Yes	13.09.2022<br>28.11.2023	BA.5<br>JN.1, BA.2.86.1
10546	3	Female	42	62	1519	V#1: Yes (n.i.); V#2: Yes (n.i.); V#3: Yes (n.i.)	Yes	xx.04.2022<br>14.12.2023	BA.2<br>JN.1
10562	3	Female	48	45-75	1108	V#1: BNT (B.1); V#2: BNT (B.1); V#3: BNT (B.1)	Yes	15.07.2022<br>xx.12.2023	BA.5<br>JN.1
10600	3	Female	36	65	2118	V#1: BNT (B.1); V#2: Yes (n.i.); V#3: Yes (n.i.); V#4: BNT (B.1/BA.5)	Yes	22.12.2022<br>12.12.2023	BQ.1.1, BF.7<br>JN.1

Cohort 1: No Infection[c]/XBB.1.5 booster; Cohort 2: One/two infection/no XBB.1.5 booster (pre-KP.2 wave);

[a]: For samples without information on the exact date of infection/vaccination a range is provided.
[b]: Anti-SARS-CoV-2 S1 IgG titers were determined against ancestral SARS-CoV-2.
[c]: SARS-CoV-2 infection-free status of cohort 1 was confirmed by ELISA (= anti-NCP IgG-negative).

Abbreviations: AZD, AZD1222/Vaxzevria; BNT (B.1), BNT162b2/Comirnaty; BNT (B.1/BA.5), Comirnaty Original / Omicron BA.4-5; BNT (XBB.1.5) Comirnaty XBB.1.5; MOD (B.1), Spikevax; RU, relative units; ID, identifier; IgG, immunoglobulin G; V#, vaccination; n.a., not applicable; n.i., no information available.
