# Supplementary figures and images for: Comparative Analysis of Host Cell Entry Efficiency and Neutralization Sensitivity of Emerging SARS-CoV-2 Lineages KP.2, KP.2.3, KP.3, and LB.1
Source: Vaccines (Basel). 2024 Oct 30;12(11):1236. doi: 10.3390/vaccines12111236 (PMC11598761; doi:10.3390/vaccines12111236)

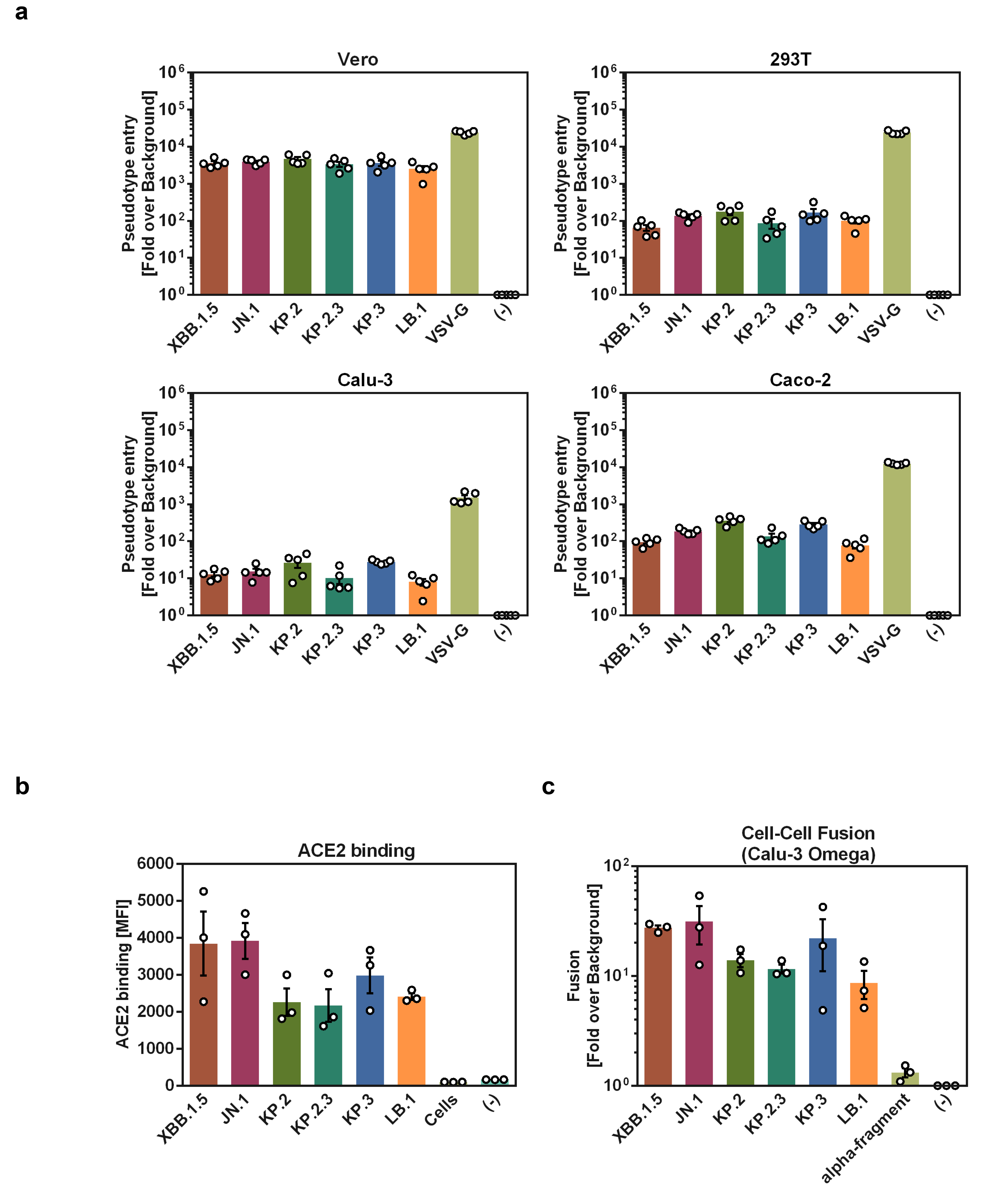

Supplement: Supplementary file 1 [file vaccines-12-01236-s001.zip › SI Figure S1.tif]

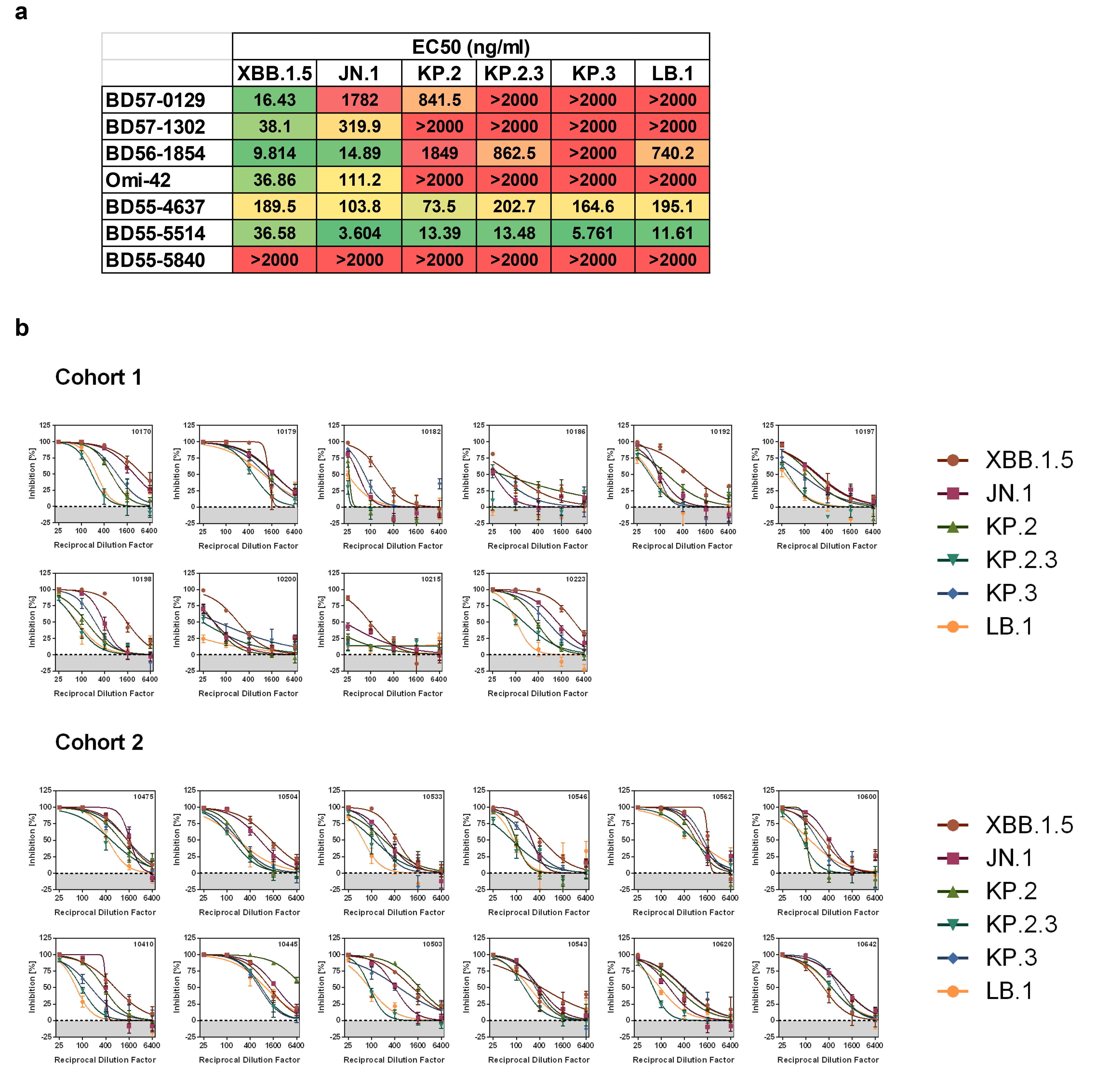

Supplement: Supplementary file 1 [file vaccines-12-01236-s001.zip › SI Figure S2.tif]
